# Assessment and management of pain in newborns hospitalized in a Neonatal Intensive Care Unit: a cross-sectional study^1^

**DOI:** 10.1590/1518-8345.1665.2931

**Published:** 2017-09-12

**Authors:** Natália Pinheiro Braga Sposito, Lisabelle Mariano Rossato, Mariana Bueno, Amélia Fumiko Kimura, Taine Costa, Danila Maria Batista Guedes

**Affiliations:** 2 MSc, RN, Hospital Universitário, Universidade de São Paulo, São Paulo, SP, Brazil. Professor, Universidade Cidade de São Paulo, São Paulo, SP, Brazil.; 3 PhD, Professor, Escola de Enfermagem, Universidade de São Paulo, São Paulo, SP, Brazil; 4 MSc, RN, Hospital Infantil Waldemar Monastier, Campo Largo, PR, Brazil.; 5 Doctoral student, Escola de Enfermagem, Universidade de São Paulo, São Paulo, SP, Brazil. Scholarship holder at Coordenação de Aperfeiçoamento de Pessoal de Nível Superior (CAPES), Brazil.

**Keywords:** Pain Measurement, Pain Management, Infant, Newborn, Neonatal Nursing

## Abstract

**Objective:**

to determine the frequency of pain, to verify the measures adopted for pain relief during the first seven days of hospitalization in the Neonatal Intensive Care Unit and to identify the type and frequency of invasive procedures to which newborns are submitted.

**Method:**

cross-sectional retrospective study. Out of the 188 hospitalizations occurred during the 12-month period, 171 were included in the study. The data were collected from the charts and the presence of pain was analyzed based on the Neonatal Infant Pain Scale and on nursing notes suggestions of pain. For statistical analysis, the Statistical Package for the Social Sciences was used, and the significance level was set at 5%.

**Results:**

there was at least one record of pain in 50.3% of the hospitalizations, according to the pain scale adopted or nursing note. The newborns underwent a mean of 6.6 invasive procedures per day. Only 32.5% of the pain records resulted in the adoption of pharmacological or non-pharmacological intervention for pain relief.

**Conclusion:**

newborns are frequently exposed to pain and the low frequency of pharmacological or non-pharmacological interventions reinforces the undertreatment of this condition.

## Introduction

Painful experiences in the neonatal period may result in physiological and behavioral alterations, as well as changes in the development of the nervous system, which can provoke considerable damage in the future^1^ ^-^ ^2^. However, several studies indicate that hospitalization in a Neonatal Intensive Care Unit (NICU) includes a high number of painful procedures^3^ ^-^ ^5^, most of them necessary for diagnosis and treatment.

Moreover, studies still show gaps in the knowledge of nursing professionals regarding assessment and management of pain^6^ ^-^ ^7^. In addition, a research demonstrates that, in general, the use of the available analgesic treatments is inadequate and insufficient^7^. Another study emphasizes that a considerable number of health professionals do not assess the level of pain based on scales developed for this purpose^8^. This result demonstrates the need to increase the use of the available evidence on effective measures for pain management, in order to improve the care provided to newborns (NBs).

Faced with this alarming reality regarding neonatal pain, the objectives of this study were to determine the frequency of pain, to verify the measures adopted for pain relief during the first seven days of hospitalization in a NICU and to identify the type and frequency of invasive procedures to which newborns are submitted.

## Method

This is a retrospective and cross-sectional study carried out at the NICU of a medium-complexity public teaching hospital located in the city of São Paulo, Brazil. The sample population was composed by NBs hospitalized in this unit. In this study, the conditions that could be related to pain, such as the medical devices in use and the painful procedures to which the newborns were submitted to during the first seven days of hospitalization were considered. 

Regarding the term “devices in use”, all devices used in the NBs for therapeutic or monitoring purposes, such as catheters, drains, cannulas and rectal thermometers, were listed. Regarding the term “invasive procedures”, it should be noted that the definition used is based on an previous study^9^. Therefore, were considered as invasive those procedures that affected the integrity of the skin or mucosa, as well as those in which there was insertion of devices in natural cavities.

The inclusion criteria adopted were: newborns admitted to the NICU between June 2013 and May 2014; and, at the moment of admission, the maximum postnatal age was 28 days for the full-term newborns (FTNB) and for the preterm newborns (PTNB) the maximum postmenstrual age (PMA) was 44 weeks. NBs who were hospitalized for less than 6 hours and those who, at admission, were older than 28 days or had a PMA of 45 weeks or more were excluded.

During the collection period, 188 hospitalizations occurred in the NICU. Of these, 17 were excluded: two because they were not located by the Medical Records and Statistics Service after several attempts; five because the hospitalization period was shorter than six hours; and 10 because the newborns were older than 28 days at the time of admission. It is worth noting that, of the total of 171 eligible hospitalizations, 21 consisted of rehospitalizations of NBs previously included in the study. These data were considered because each hospitalization resulted in a new clinical and care scenario, with its own painful events. Therefore, the data of this study refer to 171 hospitalizations corresponding to 150 NBs. 

For data collection, the medical and nursing records on the NBs files were read and an instrument elaborated by the authors, consisting of three parts, was used. The first part refers to the anthropometric data, delivery and hospitalization diagnosis; the second, to invasive procedures performed, ventilation, devices and medications in use; and the third, to the application of the Neonatal Infant Pain Scale (NIPS), nursing notes indicating pain, and the pharmacological or non-pharmacological interventions performed up to one hour after the record of pain. 

The pharmacological measures were the use of analgesic or sedative medication and anesthetics; and non-pharmacological measures were all the interventions described in the nursing notes, provided they were indicated as related to the record of pain. 

The nursing team of the institution where the research was conducted uses the NIPS scale for daily pain assessment in NBs since 2011. The NIPS is an instrument created in 1993 to assess the level of pain in full and preterm newborns. Scores higher than 3 in the scale indicate the presence of pain^10^. However, for this study, pain was considered present when the score was above zero, since, according to the pain evaluation form used at the institution, a score between 1 and 2 indicates mild pain, between 3 and 5 it indicates moderate pain, and between 6 and 7, severe pain. It should be noted that although the NIPS is part of the form adopted for all patients hospitalized in the pediatric sectors, there is no routine or pre-established flowchart for pain relief measures.

Despite the fact that the institution has a scale for assessing pain, the nursing notes that described the newborn as crying, agitated or with an expression of pain were considered as an alternative source of information, since the presence of these conditions would minimally correspond to a score one on the NIPS, and due to the empirical knowledge that the application of the scale occurs less often than ideal.

The research was approved by the Research Ethics Committees of the University of São Paulo Nursing School and of the hospital assessed, under the numbers 1,024,158 and 1,064,466, respectively. Considering the characteristics of the data collection, exemption from the Consent Form (CF) was requested. 

The tabulated data were analyzed by the Statistical Package for Social Sciences (SPSS), version 22.0. The descriptive analysis of the continuous variables was performed by the study of frequencies, central tendency and dispersion measures, and the nominal variables were described in percentages. Fisher’s exact test was used to study the correlation between qualitative variables, Pearson’s correlation was used for the quantitative variables and the ANOVA model was used to analyze the correlation between qualitative and numerical variables. In addition, Odds Ratio (OR) was calculated to analyze the association between categorical variables and, for that, contingency tables were used.

## Results

Out of the 171 hospitalizations, 134 were newborns who were admitted in the NICU only once, while the remaining 37 correspond to 16 newborns that were admitted between 2 and 4 times in the period assessed. Therefore, a total of 150 NBs were included in the study. Most of the participants (56%) were male, and the mean length of stay in the NICU was 9.12 days, as shown in Table 1, which displays the main characteristics of the newborns and their hospitalizations. The records of three NBs did not include Gestational Age (GA). However, for the 147 NBs for which this data was available, PMA ranged from 23 to 43 weeks and, similarly to GA, presented a median of 36 weeks.

**Table 1:** Characteristics of newborns and their hospitalizations in the Neonatal Intensive Care Unit. São Paulo, SP, Brazil, 2013-2014

**Characteristics**	**N (%)**	**Mean (SD)**
Birth weight*		**2485.9 (930.89)**
**Length of stay in the hospital** ^†^		**9.12 (23.89)**
Gestational age at birth*		**34.6 (4.55)**
**PMA** ^‡^ **at admission** ^†^		**35.5 (4.44)**
**PMA at admission 23-27 weeks** ^†^	**9 (5.3)**	
**PMA at admission 28-33 weeks** ^†^	**47 (27.5)**	
**PMA at admission 34-36 weeks** ^†^	**31 (18.1)**	
**PMA at admission >36 weeks** ^†^	**81 (47.4)**	
**No record of GA** ^†^	**3 (1.75)**	
**Ventilatory support (CPAP** ^§^ **or mechanic)** ^†^	**141 (82.5)**	
Male*	**84 (56)**	
Born in the same institution*	**134 (89.9)**	
Adequate for the GA*	**115 (76.7)**	
Deaths*	**10 (6.7)**	

*Total of 150 NBs; †total of 171 hospitalizations; ‡PMA: postmenstrual age; §CPAP: continuous positive airway pressure

Of the 141 hospitalizations in which there was use of ventilatory support, mechanical ventilation was used in 78 (55.3%), 39 of them in alternation with CPAP. There was statistical significance in the relationship between mechanical ventilation and continuous use of analgesic or sedative (p<0.001), and mechanically ventilated NBs were 6.1 times more likely to receive continuous analgesia and 1.8 times more likely to be prescribed analgesic or sedative under *Pro Re Nata* (PRN) or On Medical Criteria (OMC) regimens.

A total of 16 devices were used during the first week of hospitalization. A mean of 3.25 devices (Standard Deviation-SD of 1.34) per day of hospitalization was found, with a statistically significant relationship between number of devices and nursing notes of agitation (p=0.014) and crying (p<0.001). However, the same result was not obtained for NIPS score above zero (p=0.196).

A total of 4,765 procedures were performed, which corresponds to a median of six, a mean of 6.6 per day of hospitalization per NB, and a mean of 27.9 per hospitalization. A total of 25 different procedures were registered, among which the most common was heel stick (1,702; 36.1%), followed by aspiration of airways (1,240; 26.3%), venous puncture for collection of exams (426; 9%) and venous puncture for peripheral catheterization (344; 7.2%). 

Records of non-pharmacological interventions related to the performance of the procedures were not found in the medical records and no pharmacological intervention was registered for more than 96% of the total number of procedures. Of the 172 (3.6%) procedures in which at least one analgesic or sedative was used, the most frequent interventions were the combination of midazolam and Fentanyl® (37.8%) and the administration of midazolam alone (33.9%).

There was a statistically significant association between the number of procedures and the number of devices in use (p<0.001; r=0.528) and a statistically significant inverse association with the day of hospitalization (p<0.001; r=-0.248), according to Pearson’s correlation. However, the number of procedures was not statistically significant when correlated to PMA (P=0.685, r=-0.015) and birth weight (p=0.283, r=0.040).

As shown in Table 2, the qualitative variables that had a statistically significant association with the number of procedures were: spontaneous and mechanical ventilation, use of analgesic or sedative under continuous, intermittent, PRN or OMC regimen, NIPS above zero, and record of crying in the nursing notes.

**Table 2:** Description of mean, median, standard deviation and correlation between the variables and the number of invasive procedures performed on newborns admitted to the Neonatal Intensive Care Unit. São Paulo, SP, Brazil, 2013-2014

Variables			Invasive procedures			F	P value^**†**^
			Mean	SD*	Median		
Ventilation							
	**Spontaneous**					**170.8**	**<0.001**
		**Yes**	**4.60**	**3.62**	**4**		
		**No**	**8.60**	**4.52**	**8**		
	**CPAP** ^**‡**^					**1.4**	**0.231**
		**Yes**	**6.89**	**3.95**	**6**		
		**No**	**6.47**	**4.92**	**5**		
	**Mechanical**					**334.2**	**<0.001**
		**Yes**	**10.25**	**4.52**	**10**		
		**Noo**	**4.81**	**3.33**	**4**		
NIPS^****‡****^ >0						**7.1**	**0.008**
		**Yes**	**7.90**	**4.86**	**7**		
		**No**	**6.50**	**4.49**	**6**		
Nursing notes							
	**Agitation**				**6**	**0.8**	**0.377**
		**Yes**	**7.10**	**4.25**	**6**		
		**No**	**6.59**	**4.59**	**6**		
	**Crying**					**4.1**	**0.044**
		**Yes**	**5.57**	**4.14**	**4**		
		**No**	**6.75**	**4.59**	**6**		
	**Expression of pain**					**0.8**	**0.809**
		**Yes**	**7.00**	**2.92**	**8**		
		**No**	**6.63**	**4.58**	**6**		
Use of analgesic or sedative regardless of procedures performed							
	**Continuous**					**125.4**	**<0.001**
		**Yes**	**10.77**	**4.93**	**10**		
		**No**	**5.89**	**4.07**	**5**		
	**Intermittent**					**5.5**	**0.019**
		**Yes**	**7.60**	**4.53**	**7**		
		**No**	**6.47**	**4.55**	**6**		
	**PRN** ^**||**^ **ou OMC** ^**¶**^					**90.7**	**<0.001**
		**Yes**	**8.62**	**4.84**	**8**		
		**No**	**5.46**	**3.95**	**5**		
Specific non-pharmacological intervention						**0.129**	**0.720**
		**Yes**	**7.43**	**4.65**	**6**		
		**No**	**6.80**	**4.55**	**6**		
Specific pharmacological intervention							
		**Yes**	**6.91**	**4.44**	**6**	**0.018**	**0.893**
		**No**	**6.80**	**4.63**	**6**		

*SD: standard-deviation; † ANOVA model; ‡CPAP: Continuous Positive Airway Pressure; §NIPS: Neonatal Infant Pain Scale*;* ||PRN: *Pro Re Nata*; ¶OMC: on medical criteria.

There was a total of 3,884 records of the application of the NIPS for pain assessment in the NBs hospitalized in the NICU during the total of 718 days of hospitalization, which indicate a mean of 5.4 records per day of hospitalization. Of this total, 96.8% corresponded to the absence of pain, and of the remaining 123 (3.2%), 102 (82.9%) scored between 1 and 3, and 21 (17.1%) between 4 and 7. Only three of the NIPS applications consisted of evaluations after performing procedures and 11 were related to reassessment after intervention for pain relief.

There were 237 applications of the NIPS with a score above zero and/or nursing note suggesting pain: 114 were nursing notes, 102 NIPS scores and 21 consisted of a simultaneous record of both on the same day and time. This total corresponds to 86 admissions: in 18 there was only a nursing record suggesting pain; in 34 only a NIPS above zero was observed and in 34 both were recorded. In summary, in 50.3% of hospitalizations there was at least one record indicating pain during the hospitalization period.

Table 3 shows the frequency of pharmacological and non-pharmacological interventions, as well as the combination of both, according to NIPS score and presence of nursing note suggesting pain.

**Table 3:** Frequency and type of intervention for pain relief according to type of record. São Paulo, SP, Brazil, 2013-2014

Type of record		Type of intervention								No intervention		Total
		Pharmacological			Non-pharmacological			Pharmacological and non-pharmacological				
		n	%		n	%		n	%			
NIPS*												
	**Score 1 to 3**	**13**	**14.8**		**2**	**2.3**		**-**	**-**	**73**	**82.9**	**88**
	**Score 4 to 7**	**1**	**7.15**		**1**	**7.15**		**-**	**-**	**12**	**85.7**	**14**
Nursing note												
	**Crying**	**2**	**7.4**		**1**	**3.7**		**-**	**-**	**24**	**88.9**	**27**
	**Agitation**	**15**	**65.2**		**-**	**-**		**1**	**4.4**	**7**	**30.4**	**23**
	**Expression of pain**	**4**	**80.0**		**-**	**-**		**-**	**-**	**1**	**20.0**	**5**
	**Crying + agitation**	**11**	**19.6**		**6**	**10.7**		**2**	**3.6**	**37**	**66.1**	**56**
	**Agitation + expression of pain**	**3**	**100.0**		**-**	**-**		**-**	**-**	**-**	**-**	**3**
NIPS* + nursing note												
	**Score 1 to 3**	**7**	**50**		**2**	**14.3**		**-**	**-**	**5**	**35.7**	**14**
	**Score 4 to 7**	**3**	**42.8**		**2**	**28.6**		**1**	**14.3**	**1**	**14.3**	**7**
Total		**59**	**24.9**		**14**	**5.9**		**4**	**1.7**	**160**	**67.5**	**237**

*NIPS: Neonatal Infant Pain Scale.

The record of NIPS score above zero presented a statistically significant association with records of agitation and crying (p<0.001). The same did not occur with records of expression of pain (p=0.300). The simultaneous presence of nursing record suggesting pain and NIPS score above zero resulted in a 10.4 OR for pharmacological intervention, a value calculated based on the contingency table. In addition, as it can be seen in Table 4, NIPS scores above zero did not show a statistical association with any type of intervention, and only records of agitation and crying showed a statistically significant relationship with pharmacological interventions.

**Table 4:** Estimates of the association between pharmacological or non-pharmacological interventions on neonates hospitalized in a Neonatal Intensive Care and Neonatal Infant Pain Scale scores or nursing notes of agitation, crying or expression of pain. São Paulo, SP, Brazil, 2013-2014

Variables		Pharmacological intervention					Non-pharmacological intervention			
		OR*	CI 95%^**†**^	p-value^**‡**^	SE^**§**^		OR*	CI 95%^**†**^	p-value^**‡**^	SE^**§**^
NIPS^||^										
	**NIPS** ^**||**^ **>0**	**1.15**	**0.56-2.34**	**0.721**	**1.44**		**4.92**	**0.58-41.95**	**0.138**	**2.98**
Nursing note										
	**Agitation**	**2.69**	**0.77-9.44**	**<0.001**	**1.48**		**1.67**	**0.36-7.75**	**0.701**	**2.19**
	**Crying**	**1.20**	**0.59-2.43**	**0.618**	**1.43**		**1.67**	**0.36-7.75**	**0.701**	**2.19**
	**Expression of pain**	**5.32**	**1.26-22.33**	**0.021**	**2.08**		**1.06**	**0.05-20.12**	**1.000**	**4.50**

*OR: odds ratio; †CI: confidence interval; ‡Fisher’s Exact Test; §SE: standard error; ||Neonatal Infant Pain Scale.

Regarding the prescription and administration of sedatives and analgesics within a period of up to 1 hour after a NIPS score above zero or a nursing record suggesting pain, a total of 157 prescriptions were observed, of which 63 (40.1%) resulted in its administration. Midazolam, dipyrone and chloral hydrate were the drugs with the highest frequencies of prescription, respectively 31.8%, 31.2% and 20.4%. However, only 28.6% of the prescriptions of dipyrone and 36% of the prescriptions of midazolam resulted in its administration. Therefore, the drugs with the highest frequencies of administration were: morphine (100%), chloral hydrate (65.6%), Tramal® and propofol (50%).

The 21 non-pharmacological interventions correspond to 18 hospitalizations. Non-nutritive sucking and holding in ventral position were the most frequent interventions (5; 24%), followed by swaddling (3; 14%), comfort and touch (2; 9%), kangaroo care, tucking, nurturance and holding the baby (1; 5%). 

## Discussion

The inverse association found between PMA and length of stay in the hospital serves as a warning to incite the development of institutional policies addressing the particularities of premature infants and the demands of their caregivers, who often experience progress and setbacks during hospitalization and are left emotionally vulnerable as a result. 

On the other hand, regarding the conditions related to the presence of pain, it is worth mentioning that mechanical ventilation is one of the most common sources of chronic pain in the NICUs^2^, which contributes to the statistically significant relationship found between mechanical ventilation and continuous use of analgesic or sedative. Regarding the frequency of use of analgesia or sedation, about 67% of the newborns who were under mechanical ventilation received analgesic or sedative therapy in a continuous and/or intermittent regimen at some point, a value lower than the rate of 82% obtained in a study involving 243 NICUs from European countries^11^. This study found that NBs required a longer time of mechanical ventilation when compared to the other patients^11^, which demonstrates the need for an in-depth evaluation of the prescription of these drugs, considering the cost-benefit relation for each patient.

Regarding the devices used during the first week of hospitalization in the NICU, it is possible to observe in clinical practice that, in addition to the pain caused by the insertion of devices, maintaining them also bothers the NBs, mainly due to its manipulation. The statistically significant association found between the number of devices and nursing notes of agitation and crying corroborates this perception and indicates the need to develop actions aimed at ensuring the well-being of the NBs in this condition, such as the creation of specific routines and schedules for removal of catheter, in order to avoid interrupting sleep of the newborn. 

The absence of a statistically significant relations between the number of devices and NIPS score above zero can be explained by the fact that the scale is applied at standard hours, usually once or twice per period, unlike the nursing notes. The results of this study show that the application of the scale was limited to the schedules pre-established and it was rarely used in face of the painful events experienced or for the reassessment of pain. This demonstrates the need to raise awareness among nursing professionals about the role of this instrument as part of the care provided.

Besides the devices used, the procedures experienced during hospital stay are also an important and well-known factor related to pain. A systematic review with 18 observational studies on procedural pain in neonates admitted to NICU found values varying between 7.5 and 17.3 invasive procedures per neonate per day of hospitalization^3^. These values ​​are higher than the mean of 6.6 found in this study. The considerable variation between the values described in the literature may be explained by the differences between the countries where the studies were conducted, regarding economic development, research centers of and diffusion of knowledge. The variation can also be related to the different methodological designs of the studies included. It should be noted that only four of the studies included were retrospective, category in which the present study fits.

Despite the high frequency of application of the NIPS in the NICU assessed, less than 4% of the total were scores indicating pain. This data probably does not accurately reflect the conditions experienced by the NBs, considering the high number of procedures and devices in use. Therefore, this result may indicate failures and difficulties in the process of implementation or application of the scale. A Brazilian study conducted with professionals working in a neonatal unit in the Central-West Region found that, although most of the professionals reported knowing a pain assessment scale, only 24% reported using it at all times^12^. Therefore, it is necessary to investigate the personal, structural and organizational barriers that prevent or hamper the application of the knowledge acquired by professionals.

The statistically significant association found between the number of procedures and NIPS score above zero was expected, considering the procedural pain involved. However, the analysis of the very low rate of pharmacological interventions specifically related to the performance of the procedures indicates that the use of these drugs was restricted to more invasive procedures or those that can be performed more quickly and with less risks if the NB is calm, such as pleural tap, peritoneal dialysis and intubation, which presented rates of sedation and analgesia higher than 70%. In addition, the frequency of administration of midazolam alone associated with procedures (33.9%) is alarming, since this drug does not provide analgesia.

Considering these observations, the relationship between the number of procedures and analgesia or sedation in continuous, intermittent, PRN or OMC regimen seems to be influenced not only by the intention to relieve or reduce pain, but also by the easiness the sedation provides for the procedures. Consequently, health professionals must ask who and what is the focus of the care provided, so that they can put the patient as the center of care.

The absence of non-pharmacological interventions specifically related to the procedures performed is alarming, especially regarding heel stick and aspiration of airways, since these procedures are painful and occur frequently in the NICU. Also, the easiness of the measures recommended, such as sweet solutions, kangaroo care and swaddling, should be highlighted^13^ ^-^ ^14^.

In 50.3% of the hospitalizations, the newborns presented pain at least once during hospitalization. This frequency is higher than the value of 30%^15^ obtained from pediatric patients’ charts in another study. However, it still is probably underestimated, since this study indicated the existence of documentation gaps, seeing that the frequency of pain was 72% when it was reported by the nurse, the patient, or the caregivers.

Interventions were registered in only 32.5% of the cases in which pain was recorded according to the NIPS score or nursing notes, and the simultaneous presence of both sources increased the chance of the NB receiving the intervention. Therefore, it is possible to question if nursing professionals value this scale as an isolated method of pain identification. Moreover, the absence of a statistically significant correlation between sedation or analgesia and NIPS scores above zero leads to the understanding that the presence of pain according to the scale was not a parameter that led to higher rates of pharmacological interventions.

According to Brazilian studies, crying and facial expression are the main parameters used for pain assessment^6^ ^-^ ^7^ ^,^ ^16^. However, using crying as an indicator of pain is difficult, not only because it occurs in situations where there is no painful stimulus, but also because the NBs might be unable to cry due to the devices used or their health conditions^17^. Therefore, the fact that, in this study, the isolated record of crying resulted in the smallest number of interventions may be due to its non-specificity or because it is not considered a condition that alone justifies an intervention.

Still on the low frequency of interventions for pain relief, it is worth noting that non-pharmacological interventions are recognized as effective when isolated or as measures complementary to pharmacological treatment^2^ ^,^ ^12^ ^-^ ^13^ ^,^ ^18^. Also, they are a potential field of action for nursing care, and have not been properly implemented yet, considering the frequencies described^3^ ^-^ ^5^. Consequently, it is necessary to overcome the existing barriers, promoting knowledge about the subject and autonomy for decision making. 

Therefore, it is necessary that nursing professionals use this evaluation for implementing actions and elaborating strategies for permanent education on neonatal pain and for raising awareness about the importance of recording the activities performed and incorporating the role of supervision of care.

However, it is necessary to consider the possibility that the data related to these care actions may be impaired by the lack of records, since, according to two Brazilian studies carried out with nursing professionals working in neonatal units in the Southeast and Northeast Regions, a considerable proportion of the participants reported they did not or rarely registered the non-pharmacological measure adopted, with frequencies around 50%, among the nurses, and around 20% among the nursing assistants/technicians in both studies^6^ ^-^ ^7^.

The discrepancy between identification and management of pain can occur for several reasons. Professionals working in Canadian NICUs have identified three themes that influence pain-related practice: a culture of collaboration and support for evidence-based practice, threats to autonomous decision-making, and the complexities in care delivery^19^. Inter-professional collaboration and trust, joint work with families and the incentive for professional development were considered favorable situations. On the other hand, hierarchical relationships, care based on personal preferences, patient-related complexities, and organizational culture were unfavorable factors to the quality of care^19^.

 The lack of changes in the context of the culture of pain goes beyond borders and requires the participation and joint action of managers of health organizations, professionals at all levels of care, and family members^18^.

## Conclusion

The data presented indicate undertreatment of pain and underutilization of the NIPS as an instrument to guide nursing care for pain management. The presence of pain was recorded in approximately half of the hospitalizations through the NIPS score or the nursing notes. Regarding the procedures, it was observed that the newborns are exposed to a large amount and diversity of invasive procedures during the hospitalization, especially heel stick and aspiration of airways. 

A significant deficiency of pharmacological and non-pharmacological interventions for effective pain relief is noted, since more than half of the records of pain did not result in the adoption of any measure. In the hospitalizations where they were adopted, pharmacological interventions were more frequent.

As limitations of the study, it is important to note that it was conducted in a single institution and it had a retrospective design. Therefore, health professionals’ records probably do not accurately reflect the care provided. Thus, future studies should cover a larger number of institutions in order to allow the comparison of assessment and management of neonatal pain in different scenarios, and also use prospective designs as a way to minimize data loss. 

## References

[1] Valeri BO, Holsti L, Linhares MBM (2015). Neonatal Pain and Developmental outcomes in children born preterm a
systematic review. Clin J Pain.

[2] Hall RW, Anand KJS (2014). Pain management in newborns. Clin Perinatol.

[3] Cruz MD, Fernandes AM, Oliveira CR (2016). Epidemiology of painful procedures performed in neonates a systematic
review of observational studies. Eur J Pain.

[4] Kyololo OM, Stevens B, Gastaldo D, Gisore P (2014). Procedural pain in neonatal units in Kenya. Arch Dis Child Fetal Neonatal.

[5] Roofhooft DWE, Simons SH, Anand KJ, Tibboel D, van Dijk M (2014). Eight years later, are we still hurting newborn infant. Neonatology.

[6] Soares ACO, Caminha MFC, Coutinho ACFP, Ventura CMU (2016). Dor na Unidade Neonatal: conhecimento, atitude e prática da equipe de
enfermagem. Cogitare Enferm.

[7] Christoffel MM, Castral TC, Daré MF, Montanholi LL, Gomes ALM, Scochi CGS (2017). Atitudes dos profissionais de saúde na avaliação e tratamento da dor
neonatal. Esc Anna Nery.

[8] Nimbalkar AS, Dongara AR, Phatak AG, Nimbalkar SM (2014). Knowledge and attitudes regarding neonatal pain among nursing staff of
pediatric department an Indian experience. Pain Manag Nurs.

[9] Carbajal R, Rousset A, Danan C, Coquery S, Nolent P, Ducrocq S (2008). Epidemiology and treatment of painful procedures in neonates in
Intensive Care Units. JAMA.

[10] Lawrence J, Alcock D, McGrath P, Kay J, MacMurray SB, Dulberg C (1993). The development of a tool to assess neonatal pain. Neonatal Netw.

[11] Carbajal R, Ericksson M, Courtois E, Boyle E, Avila-Alvarez A, Andersen RD (2015). Sedation and analgesia practices in neonatal intensive care units
results from a prospective cohort study. Lancet Respir Med.

[12] Oliveira IM, Castral TC, Cavalcante MMFP, Carvalho JC, Daré MF, Salge AKM (2016). Conhecimento e atitude dos profissionais de enfermagem sobre avaliação
e tratamento da dor neonatal. Rev Eletr Enferm.

[13] Johnston C, Campbell-Yeo M, Disher T, Benoit B, Fernandes A, Streiner D (2017). Skin-to-skin care for procedural pain in neonates. Cochrane Database Syst Rev.

[14] Committee On Fetus And Newborn And Section On Anesthesiology And Pain
Medicine (2016). Prevention and management of procedural pain in the neonate: an
Update. Pediatrics.

[15] Shomaker K, Dutton S, Mark M (2015). Pain Prevalence and treatment patterns in a US Children's
Hospital. Hosp Pediatr.

[16] Santos MCC, Gomes MFP, Capellini VK, Carvalho VCS (2015). Avaliação materna da dor em recém-nascidos prematuros. Rev Rene.

[17] Hatfield LA, Ely AE (2015). Measurement of acute pain in infants: a review of behavioral and
physiological variables. Biol Res Nurs.

[18] Harrison D, Bueno M, Reszel J (2015). Prevention and management of pain and stress in the
neonate. Res Rep Neonatol.

[19] Stevens B, Riahi S, Cardoso R, Ballantyne M, Yamada J, Beyene J (2011). The influence of context on pain practices in the NICU perceptions of
health care professionals. Qual Health Res.

